# Effect of traditional Chinese medicine in osteosarcoma: Cross-interference of signaling pathways and potential therapeutic targets

**DOI:** 10.1097/MD.0000000000036467

**Published:** 2024-01-19

**Authors:** Yuezhen Liu, Bing Jiang, Yanqiang Li, Xiaoshou Zhang, Lijun Wang, Yasai Yao, Baohong Zhu, Hengwei Shi, Xiping Chai, Xingrong Hu, Bangneng Zhang, Hongzhuan Li

**Affiliations:** a Clinical College of Traditional Chinese Medicine, Gansu University of Traditional Chinese Medicine, Lanzhou, China; b Department of Integrated Chinese and Western Medicine, Gansu University of Traditional Chinese Medicine, Lanzhou, China; c The Second Affiliated Hospital of Gansu University of Traditional Chinese Medicine, Lanzhou, China; d Gansu Provincial Hospital of Traditional Chinese Medicine, Lanzhou, China.

**Keywords:** osteosarcoma, traditional Chinese medicine, signaling pathway, potential target, ferroptosis

## Abstract

Osteosarcoma (OS) has a high recurrence rate, disability rate, mortality and metastasis, it brings great economic burden and psychological pressure to patients, and then seriously affects the quality of life of patients. At present, the treatment methods of OS mainly include radiotherapy, chemotherapy, surgical therapy and neoadjuvant chemotherapy combined with limb salvage surgery. These treatment methods can relieve the clinical symptoms of patients to a certain extent, and also effectively reduce the disability rate, mortality and recurrence rate of OS patients. However, because metastasis of tumor cells leads to new complications, and OS cells become resistant with prolonged drug intervention, which reduces the sensitivity of OS cells to drugs, these treatments still have some limitations. More and more studies have shown that traditional Chinese medicine (TCM) has the characteristics of “multiple targets and multiple pathways,” and can play an important role in the development of OS through several key signaling pathways, including PI3K/AKT, Wnt/β-catenin, tyrosine kinase/transcription factor 3 (JAK/STAT3), Notch, transforming growth factor-β (TGF-β)/Smad, nuclear transcription factor-κB (NF-κB), mitogen-activated protein kinase (MAPK), nuclear factor E2-related factor 2 (Nrf2), Hippo/YAP, OPG/RANK/RANKL, Hedgehog and so on. In this paper, the signaling pathways of cross-interference between active ingredients of TCM and OS were reviewed, and the development status of novel OS treatment was analyzed. The active ingredients in TCM can provide therapeutic benefits to patients by targeting the activity of signaling pathways. In addition, potential strategies for targeted therapy of OS by using ferroptosis were discussed. We hope to provide a unique insight for the in-depth research and clinical application of TCM in the fields of OS growth, metastasis and chemotherapy resistance by understanding the signaling crosstalk between active ingredients in TCM and OS.

## 1. Introduction

Osteosarcoma (OS) is the most common primary malignant bone tumor in adolescents and children.^[1]^ According to research studies, 20,000 to 30,000 patients worldwide are afflicted with osteosarcoma each year.^[2]^ Clinically, OS can be intervened by radiotherapy. Although this treatment is effective to some extent, and the 5-year survival rate of osteosarcoma patients can reach 60% to 70%, this survival rate accounts for only 20% for patients with metastatic OS,^[3]^ of which the lungs account for 90% of metastatic sites and other bones account for 8% to 10%.^[4]^ Meanwhile, radiation can also damage the normal tissue around the tumor, resulting in inevitable complications. In recent years, chemotherapeutic drugs for the treatment of OS can include methotrexate, doxorubicin, cisplatin, eflornithine and etoposide. Although these drugs have a certain significant effect on patients with OS, long-term use will lead to drug resistance in tumor cells.^[5]^ In addition, radical resection and limb salvage surgery are more than 90% of surgical cases. Although surgery can significantly improve the patient’s condition, the low number of cases makes it challenging to recruit sufficient populations to support high-quality studies,^[6]^ and the survival rate of OS patients through amputation is only 20% to 30%.^[7]^ Notably, OS patients also have a significant economic burden when faced with other complications.^[8]^ Therefore, exploring an efficient and safe strategy to treat OS is still an important topic and hotspot in the field of current medicine.

Previous studies have pointed out^[9–11]^ that traditional Chinese medicine (TCM) can regulate Wnt/β-catenin, tyrosine kinase/transcription factor 3 (JAK/STAT3), Notch, transforming growth factor-β (TGF-β)/Smad, nuclear transcription factor-κB (NF-κB), mitogen-activated protein kinase (MAPK), nuclear factor E2-related factor 2 (Nrf2), Hippo/YAP, OPG/RANK/RANKL, Hedgehog, iron death and other signaling pathway, so as to inhibit the proliferation, differentiation, metastasis of OS cells and promote the occurrence and development of OS by regulating the ratio of pro-apoptotic proteins and anti-apoptotic proteins, inhibiting the release and activity of inflammatory factors, regulating the metabolism of abnormal energy, reducing oxidative stress, improving the tumor microenvironment, accelerating the autophagic death of OS cells, inhibiting angiogenesis, and ultimately hindering the development of OS cells. In recent years, a large number of studies at home and abroad have confirmed that TCM has the characteristics of “simplicity, convenience, cheapness and superiority,” and its rich monomers and active components of TCM can induce OS cell apoptosis by regulating related signaling pathways, especially the regulatory mechanism of iron death, and then inhibit the growth and metastasis of OS, and even have the effect of reversal in acquired drug resistance. Therefore, this review mainly summarizes the regulatory effects of TCM monomers and active components on OS-related signaling pathways in order to provide reference and ideas for TCM treatment of OS, while providing a theoretical basis for subsequent deep study and clinical treatment.

## 2. Understanding of OS with TCM

In the traditional medical books of TCM, there is no record of the disease name of “OS.” According to the clinical manifestations (including swelling, pain, limited functional activity of the surrounding joints, claudication of the lower limbs and generalized fever at its lesion site), it is attributed to the categories of “lower stone melioidosis,” “bone melioidosis,” “bone tumor,” “bone erosion” and “cancer” in TCM. As recorded in the Yellow Emperor’s Internal Classic · Lingshu · Carbuncle: It occurs in the knee and is called flaw carbuncle, which is large in shape, unchanged in carbuncle color, cold and heat, such as firm stone.^[12]^

Starting from the overall concept of TCM, it is believed that the main pathogenesis of osteosarcoma is based on deficiency of vital qi, which is caused by internal injury of 7 emotions or exogenous 6 evils, resulting in qi stagnation, blood stasis, phlegm-dampness, endogenous evil toxin, remaining in the bones to give birth to a mass, and causing osteoma over time. As recorded in the Yellow Emperor ‘s Internal Classic · Lingshu · thorn section true evil: The virtual evil enters the body and is also deep, cold and heat fight, long stay and internal, cold victory its heat, then bone pain meat dry, heat victory its cold, then rotten meat rot muscle for pus, internal injury bone, internal injury bone for bone erosion.^[13]^

The etiology of OS can be divided into internal causes, external causes, and no internal and external causes. Internal causes are factors from the inside of the body, including personal physical fitness, mental status, age, genetics and other factors. Exogenous factors are the invasion of the body to feel exogenous pathogens, which are also known as “six evils” or “grumpy qi,” including 6 evils (such as wind, cold, heat, dampness, dryness, and fire). Exogenous pathogens are the main pathogenic factors of exogenous diseases in the body and are closely related to the occurrence of OS. As recorded in the Lingzhu Classic: “Ying Wei Ba,” “Cold evil guest in the extraintestinal and Wei Qi fight,” “evil residence period” and so on. In the today’s society, the so-called external factors should also include environmental pollution, biological viruses, chemical pollution, and various physical radiation and other adverse factors that damage the body beyond the scope of human health. In addition, internal and external causes are also one of the important causes of OS. The human body is often injured by 7 emotions, including anger, joy, thought, sadness, fear, shock and worry due to congenital deficiency, dietary preference, preference for fat and sweet taste, or internal and external effects such as violence and golden stone trauma, resulting in dysfunction of the viscera of the human body, meridian obstruction, stagnation and congestion, and stagnation of qi and blood, resulting in the generation of bone tumors. As recorded in the Song Dynasty “San Ji Tong Lu”: Qi and blood epidemic does not lose its constant, then the body is peaceful, no or more vegetation and stagnation congestion, then take advantage of the virtual gap, tumor so born.

## 3. OS-associated signaling pathway

### 3.1. PI3K/Akt signaling pathway

PI3K/Akt is a highly conserved signaling pathway, which mainly involved in processes of cell growth, proliferation, apoptosis, and metabolism. PI3K is an intracellular phosphatidylinositol kinase, and PI3Ks constitute a family of lipid kinases, which can be divided into 3 classes, with type I PI3K being most closely related to tumors.^[14]^ When the PI3K signaling pathway is activated, it can bind to the downstream member Akt and keep Akt in an active state,^[15]^ while phosphorylated Akt is a key link in initiating the PI3K/Akt signaling pathway.^[16]^ Increasing evidence suggests^[13]^ that many compounds are involved in both PI3K/AKT signaling and regulation of OS metabolism, such as phosphatase and angiotensin homolog (PTEN), pro-apoptotic protein (Bax), autophagy-related protein (LC3), pro-angiogenic factor (VEGF), mammalian target of rapamycin (mTOR), and glycogen synthesis kinase-3 (GSK-3) and so on. Among them, PTEN inhibits the growth of OS cells by regulating tumor cell proliferation, Bax by regulating apoptosis, LC3 and mTOR by regulating autophagy in tumor cells, while VEGF by regulating angiogenesis.^[17]^ Previous studies have also found^[18–21]^ that PI3K, Akt, PTEN, Bax, LC3, VEGF, mTOR, GSK-3, p-PI3K, p-Akt, and p-mTOR proteins are all relevant targets on the PI3K/Akt signaling pathway, while affecting the progression of OS with the help of the conduction of this pathway.

### 3.2. Wnt/β-catenin signaling pathway

Wnt/β-catenin signaling pathway is an evolutionarily conserved pathway, which can not only control the proliferation, survival, and differentiation of stem cells, but can also regulate the homeostasis of calcium, cellular polarity and the homeostasis of adult tissue, especially in the diseases of skeletal system.^[22]^ It can be seen that this pathway plays a critical role in proliferation, apoptosis, invasion and migration of malignant and cancer stem cells.^[23,24]^ There are 2 major pathways of Wnt signaling, namely β-catenin-dependent (canonical pathway) and β-catenin-independent pathways (non-canonical pathway).^[25]^ The main molecules involved in regulating apoptosis and proliferation of OS are Wnt3a, Wnt5a, Wnt7b, Wnt9a, GSK-3β, and proto-oncogene (C-Myc). Among them, Wnt3a and GSK-3β can inhibit cell proliferation.^[26]^ Wnt5a can inhibit tumor cell immunoregulatory cytokine interleukin 6 (IL-6), proangiogenic factor interleukin 8 (IL-8), angiogenic factor VEGF, and matrix metalloproteinase-2 (MMP2) expressions. As Wang et al^[27]^ have found that Wnt5a can attenuate the invasiveness of OS cells and their metastatic ability by regulating the release of immunomodulatory and proangiogenic molecules. Regulating the transcription of Wnt7b and Wnt9a can down-regulate the expression of collagen cross-linking enzyme Loxl2, which has an inhibitory effect on the proliferation of OS cells.^[28]^

### 3.3. JAK/STAT3 signaling pathway

JAK/STAT3 is a signal transduction pathway stimulated by cytokines, which is mainly involved in various important biological processes, such as cell proliferation, differentiation, apoptosis, immune regulation and so on.^[29,30]^ There are 4 JAKs proteins, which can be divided into JAK1, JAK2, JAK3, and TYK2, while there are 7 STATs proteins, which can be divided into STAT1, STAT2, STAT3, STAT4, STAT5A, STAT5B, and STAT6.^[31]^ When cells are stimulated exogenously, JAKs proteins bind to cytokine receptors in a non-covalent manner and mediate receptor tyrosine phosphorylation, which then binds to growth factor STATs to form complexes and is transported into the nucleus as a transcription factor to play a biological role.^[32]^ Studies have shown that the main molecules involved in the pathogenesis of osteosarcoma are JAK2, TYK2, STAT1, STAT2, STAT3, and STAT5A.^[33]^ Among them, activation of JAK2 can induce proliferation of bone marrow stem cells, and mutation of TYK2 can induce malignant propagation of lymphocytes. According to a large number of in vitro and in vivo studies, STAT1 and STAT2 in the STATs family have a role against cancer stem cell propagation. Notably, STAT3 is most closely related to the development of osteosarcoma,^[34–36]^ and it can not only induce OS cell proliferation, increase drug resistance,^[37]^ but also promote angiogenesis,^[38]^ regulate the inflammatory response and immune function in OS cells.^[39]^ Moreover, Guo et al^[40]^ analyzed 98 cases obtained from OS patients, and they found that low STAT5 expression was not associated with patient age, gender, tumor location, surgical approach, tumor area, histological response and metastasis, but was closely related to the progression of OS, indicating that STAT5A may be a novel prognostic factor for OS and can be used as a molecular target for OS therapy.

### 3.4. Notch signaling pathway

Notch is a highly conserved signaling pathway that is mainly involved in proliferation, survival, apoptosis, and differentiation of cells.^[41]^ It has been shown that in mammals, Notch signaling is composed of 4 Notch receptors (Notch-1, Notch-2, Notch-3, Notch-4), 5 ligands (Delta like-1, Delta like-3, Delta like-4, Jagged1 and Jagged 2), downstream target genes and other effectors.^[42,43]^ When the Notch signaling pathway is activated, the ligand expressed on neighboring cells triggers a series of proteolysis, which translocates the receptor in the cytoplasm into the nucleus and forms a transient transcriptional complex, so as to initiate the expression of the corresponding target gene.^[44]^ Studies have shown that Notch signaling pathway plays a critical role in the development and progression of tumors.^[45]^ Over-expressed Notch-1 receptor can up-regulate the expression level of cell division cycle 20 (Cdc-20) protein in OS cells, so as to improve the viability of OS cells.^[46]^ Additionally, over-expressed Notch-3 receptor can affect the invasion and metastasis of OS by regulating the expression levels of downstream target genes Hes1 and matrix metalloenzyme 7 (MMP-7), so as to evaluate the prognosis of OS patients.^[47]^

### 3.5. TGF-β/Smad signaling pathway

The TGF-β/Smad signaling pathway is widely involved in a variety of biological functions, including cell proliferation, differentiation, survival, angiogenesis and immune surveillance,^[48]^ and plays an important role in the growth and metastasis of multiple tumors.^[49]^ TGF-β is mainly composed of TGF-β1, TGF-β2, TGF-β3, activin, nodular protein, inhibin, mullerian inhibitory substance, growth and differentiation factor, and bone morphogenetic protein.^[50]^ Among them, TGF-β1 is a key regulator of many biological processes, which is most abundantly and widely expressed in immune cells, and is most abundant in serum, mainly involved in cell differentiation, angiogenesis, immune system and inflammatory response.^[51]^ TGF-β2 acts on T cells through the immune system, which in turn mediates tumor proliferation and apoptosis.^[52]^ TGF-β3 mRNA is expressed in lymphocytes (such as CD4^+^ T cells, CD8^+^ T cells and B cells), and is mainly involved in important processes, such as immune system and inflammatory response.^[53]^ Smads proteins are downstream receptor substrates of phosphorylated TGF-β, and TGF-β1 produces corresponding biological effects by activating specific Ser residues in the C-terminal region of Smad2 and Smad3.^[54]^ Numerous studies have shown that propagation and survival of OS cells are also closely related to the TGF-β/Smad signaling pathway.^[55–59]^ For example, Lamora et al^[60]^ found that over-expressed Smad7 affected the “vicious cycle” established between tumor cells and OS through its ability to reduce OS activity, while over-expressed Smad7 in OS cells can also inhibit primary tumor metastasis to the lung in SD208 mice.

### 3.6. NF-κB signaling pathway

NF-κB is a class of regulators with multiple physiological functions that are mainly involved in regulating the metabolism, immunity and inflammation of cells,^[61,62]^ and is also closely related to the growth, metastasis and invasion of bone tumors.^[63,64]^ When most resting cells are unstimulated, NF-κB in dimeric form binds to NF-κB inhibitory protein (iκB) in the cytoplasm to form a stable trimer and remains inactive.^[65]^ However, when cells receive various external stimuli, receptors on the cell membrane translocate into the cytoplasm, and iκB is phosphorylated by binding to the iκB kinase complex, so as to activate the NF-κB signaling pathway, at which time NF-κB moves into the nucleus and induces transcription of related genes.^[66]^ Moreover, activated NF-κB signaling pathway can also induce biological reactions in related factors, such as IL-6, tumor necrosis factor-α (TNF-α), chemokines, inflammatory mediators nitric oxide (NO), prostaglandin E2, inducible cyclooxygenase-2, adhesion factors, inflammatory enzymes, immune receptors, apoptotic genes, proliferation-regulating genes, signal transduction genes, anti-oxidative stress-related genes,^[67]^ so as to further amplify the inflammatory response.^[68]^ And the long-standing inflammatory reactions will mutate tumor suppressor genes in normal cells, eventually promote tumor formation. Meanwhile, increased NF-κB activity can also promote OS cell proliferation, inhibit OS cell apoptosis, and promote the generation of collateral vessels in OS.^[69,70]^

### 3.7. MAPK signaling pathway

MAPK pathway is an important central link in multiple signaling pathways, which is mainly involved in regulating cell proliferation, differentiation, apoptosis, cycle arrest, immunity and inflammation.^[71]^ At present, isoforms of MAPK can be divided into 3 types: extracellular signal-regulated protein kinase (ERK), c-Jun NH2-terminal kinase (JNK) and p38 mitogen-activated protein kinase (p38 MAPK) according to their unique structure and function in mammals.^[72]^ Among them, ERK is mainly most closely related to tumor differentiation and growth,^[73]^ while JNKs and p38 MAPK signaling pathways play an important role in stress responses, such as inflammation and apoptosis. Under normal physiological conditions, MAPK keeps a silent state. When cells are stimulated by some factors (such as growth factors, inflammation, and oxidative stress), the MAPK signaling pathway can be activated, and the MAPK protein at this time keeps a phosphorylated state, which mediates the interaction between transforming growth factor-β-activated protein kinase 1 (TAK1)/binding protein 1 (TAB1) and p38α, which in turn leads to the activation of the p38/MAPK signaling pathway, downregulate the expression of purinergic ligand-gated ion channel 7 receptor (P2X7R), TNF-α, IL-1β factors, and inhibit the surrounding inflammatory response as well as tumor growth.^[74]^ p38/MAPK signaling pathway has also been implicated as a mediator between stress and inflammatory responses, and has dual effects on the occurrence and development of OS.^[75]^ Moreover, when MAPK signaling pathway is activated, it can also promote angiogenesis by up-regulating the expression of vascular endothelial growth factor A, which is beneficial to increase the reproductive capacity and viability of OS cells.^[76]^

### 3.8. Other signaling pathways

Some potential signaling pathways, such as Nrf2, Hippo/YAP, OPG/RANK/RANKL and Hedgehog, are closely related to the development of OS. Nrf2, as a transcription factor, belongs to the Cap “n” collar subfamily of basic leucine zipper proteins, and promotes the survival of normal cells, cancer cells, cancer stem cells (CSC) by regulating redox homeostasis, cellular metabolism and the expression of xenobiotic detoxifying enzymes.^[77]^ The Hippo/YAP signaling pathway plays an important role in organ development, cell proliferation, homeostasis and regeneration. Its core kinases (MST1/2 and LATS1/2) are not only tumor suppressors that inhibit the oncogenic function of YAP/TAZ and TEAD,^[78]^ but also play an integral role in stem and cancer cells.^[79]^ When the Hippo/YAP signaling pathway is activated, Yes-associated protein (YAP) promotes YAP phosphorylation induced by the sarcoma gene (Src) and further promotes nuclear translocation of YAP, so as to complete transcription against Hippo-YAP signaling target genes involved in cancer evolution.^[80]^ In addition, triplet osteoprotegerin (OPG), receptor activator (RANK) and ligand RANKL that promotes tumor proliferation are also closely related to the occurrence and development of OS. RANKL secreted by tumor cells can bind to receptor RANK on the surface of osteoclasts and promote osteoclasts to differentiate into mature type. At this time, mature osteoclasts will be degraded, so as to release the present factors in the matrix of bone, such as TGF-β, insulin-like growth factor and so on, which can precisely promote the proliferation of OS cells.^[81]^ Hedgehog (Hh) signaling is a conserved signaling pathway that is mainly involved in cell proliferation, differentiation, and growth,^[82]^ and is also closely related to the development of OS. When the Hh signaling pathway is activated, its ligands (such as Indian, Sonic, Desert Hedgehog and so on) bind to the corresponding receptor Smoothened, so as to weaken the inhibitory effect of Patched 1, and up-regulate the expression levels of Gliched 1/2, Myc, Cyclin D, Bcl2 and other proteins, which in turn promotes the proliferation of OS cells.^[83]^

## 4. Targeted therapy of OS by signaling cascade associated with TCM intervention

**Figure 1.**
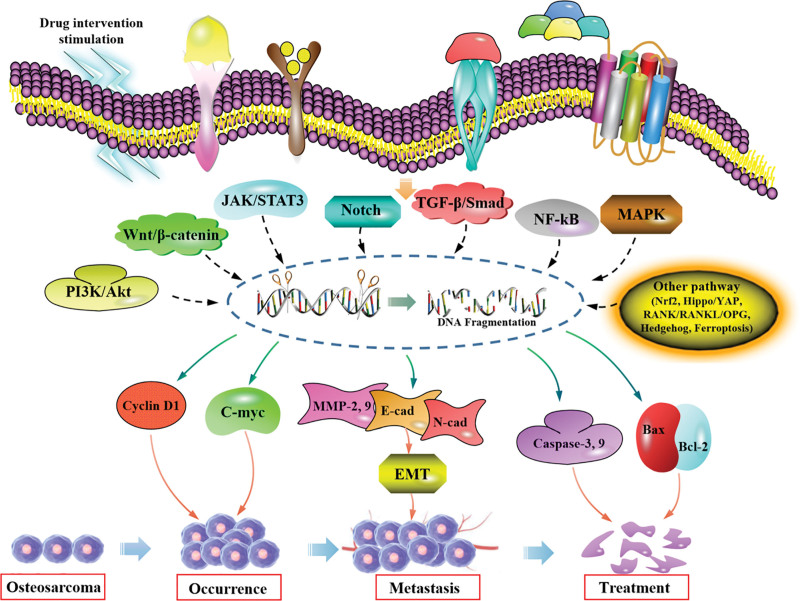
The effect of TCM on OS development and progression by regulating the activity of related signaling pathways. OS = osteosarcoma, TCM = traditional Chinese medicine.

Because signaling molecular mechanisms play an important role in regulating the development of OS, the research and development of OS drug based on the basis of signaling cascades has gradually become a research hotspot. TCM has the characteristics of “simplicity, convenience, cheapness and superiority,” and its rich TCM monomers and active components can affect the growth and metastasis of OS by regulating related signaling pathways, and even improve the acquired drug resistance of OS cells. Currently, many compounds can be extracted from TCM and their efficacy in OS can be shown through in vivo or in vitro and clinical trials. Here, we summarize these compounds to elucidate the effect of TCM on OS development and progression by regulating the activity of related signaling pathways (Fig. 1; Table 1).

**Table 1:** The active ingredient of TCM as a therapeutic strategy for OS via targeting signaling pathway.

Signaling pathway	Compound	Chinese medicine	Associated OS cell lines	References
PI3K/AKT pathway	Alantolactone	*Aucklandia lappa Decne.*	U2OS, HOS	^[84]^
	Chelerythrine	*Toddalia asiatica L.*	U2OS, MG-63	^[85]^
	Andrographolide	*Andrographis paniculata*	U2OS	^[86]^
	Rhaponticin	*Rheum palmatum L.*	MC3T3-E1, MG-63	^[87]^
Wnt/β-catenin pathway	Piperine	Piper nigrum L.	U2OS, 143B	^[88]^
	Icariin	*Epimedium brevicornu Maxim.*	143B	^[89]^
	Isoquercitrin	*Bidens pilosa L.*	143B, U2OS	^[90]^
	Polydatin	*Reynoutria japonica Houtt.*	MG-63, Saos-2	^[91]^
	Curcumin	*Curcuma longa L.*	U2OS	^[92]^
	Baicalein	*Rhizoma coptidis*	143B, MG-63, U2OS	^[93]^
	Allicin	*Allium sativum*	Saos-2, U2OS	^[94]^
	Cantharidin	*Mylabris phalerata Pallas*	U2OS, 143B, Saos-2, MG-63, MNNG	^[95]^
	Cinnamaldehyde	*Cinnamomum cassia Presl*	143B, U2OS, Saos-2, MG-63, HEB, HS5, LO2	^[96]^
	Lycorine	*Lyco salvia miltiorrhiza*	143B, MG-63, Saos-2, U2OS	^[97]^
JAK/STAT3 pathway	Notoginsenoside R1	*Panax notoginseng*	U2OS, MG-63	^[98]^
	Polydatin	*Reynoutria japonica Houtt.*	MG-63	^[99]^
	Hederoside C	*P. koreana*	MG-63, U2OS	^[100]^
	Salidroside	*Rhodiola rosea L.*	U2OS, MG-63	^[101]^
	Tubocapsenolide A	*Tubocapsicum anomalum*	SHP-2	^[102]^
	Andrographolide	*Andrographis paniculata*	U2OS	^[103]^
	Pacilitaxel	*Taxus wallichiana var. chinensis (Pilger) Florin*	U2OS, MG-63	^[104]^
	Resveratrol	*Veratrum album*	MNNG/HOS, MG-63	^[105]^
	β-caryophyllene	*Dianthus chinensis L.*	MG-63	^[106]^
	Sodium cantharidate	*Mylabris phalerata Pallas*	MG-63	^[107]^
	Tanshinone I	*Salvia miltiorrhiza Bunge*	U2OS, MOS-J	^[108,109]^
Notch pathway	Cinobufagin	*Bufo raddei*	U2OS, MG-63	^[110]^
	Curcumin	*Curcuma longa L.*	U2OS	^[92,111]^
	Cannabinoids	*Cannabis sativa L.*	MG-63	^[112]^
	Dihydroartemisinin	*Artemisia caruifolia Buch.-Ham. ex Roxb.*	U2OS	^[113]^
	Baicalein	*Scutellaria baicalensis Georgi*	143B	^[114]^
	Cordcepin	*Cordyceps*	MG-63	^[115]^
	Oleanolic acid	*Angelica sinensis (Oliv.) Diels*	U2OS, MG-63	^[116]^
	Pilose antler polypeptides	*Cartialgenous*	MG-63	^[117]^
TGF-β/Smad pathway	Oridonin	*Rabdosia rubescens (Hemsl.) Hara*	MG-63, 143B	^[118]^
	Galangin	*Alpinia officinarum Hance*	U2OS, MG-63	^[119]^
	Hyperoside	*Cuscuta chinensis Lam.*	U2OS, MG-63	^[120]^
	Dihydroxycoumarins	*Fraxetin*	U2OS, MG-63	^[121]^
	Naringenin	*Orange*	U2OS	^[122]^
	Chimaphilin	*Pyrola incarnata Fisch*	U2OS	^[123]^
	Geraniin	*Agrimonia pilosa Ledeb.*	U2OS	^[124]^
NF-κB pathway	Diosgenin	*Dioscorea polystachya Turczaninow*	U2OS	^[125]^
	Punicalagin	*Pomegranate*	HOS, U2OS, MG-63	^[126]^
	Magnoflorine	*Chelidonium majus L.*	MG-63, U2OS	^[127]^
	Theabrownin	*Camellia sinensis var. assamica*	U2OS	^[128]^
	Asiaticoside	*Centella asiatica (L.) Urb.*	THP-1, U2OS	^[129]^
	Magnolol	*Houpoea officinalis*	U2OS	^[130]^
	Paeonol	*Moutan cortex*	U2OS	^[131]^
	Sulforaphene	*Raphanus sativus L.*	U2OS, Saos-2	^[132]^
	Curculigoside	*Curculigo orchioides Gaertn.*	U2OS	^[133]^
	Dihydromyricetin	*Morella rubra Lour.*	U2OS, HOS	^[134]^
	Fistein	*Wood wax*	U2OS	^[135]^
	Ginsenoside Rh2	*Panax ginseng C. A. Mey.*	U2OS	^[136]^
MAPK pathway	Dioscin	*Dioscorea polystachya Turczaninow*	MG-63, U2OS	^[137]^
	Lycorine	*Lycoris radiata (L’Her.) Herb.*	SJSA-1, U2OS	^[97,138]^
	Delphinidin	*Consolida ajacis (Linn.) Schur*	HOS, MG-63	^[139]^
	Oleandrin	*Cuscuta chinensis Lam.*	143B, U2OS, MG-63	^[140]^
	Tanshinone	*Salvia miltiorrhiza Bunge*	U2OS, MG-63, Saos-2, 143B, G292, HU03N1	^[109,141]^
	Lentinan	*Lentinula edodes*	MG-63	^[142]^
	Berberine	*Coptis chinensis Franch.*	MG63, U2OS	^[143,144]^
	Myricetin	*Morella rubra Lour.*	D-17, DSN	^[145]^
	Quercetin	*QuercusdentataThunb.*	DSN	^[146]^
	Cardamonin	*Myristica fragrans Houtt.*	U2OS, MG-63	^[147]^
Nrf2 pathway	Tanshinone IIA	*Salvia miltiorrhiza Bunge*	MG-63	^[148]^
	Oridonin	*Rabdosia rubescens (Hemsl.) Hara*	MG-63	^[149]^
	Emodin	*Rheum palmatum L.*	MG-63	^[150]^
Hippo/YAP pathway	Norcantharidin	*Mylabris phalerata Pallas*	U2OS	^[151]^
RANK/RANKL/OPG pathway	Jialien	*Jialien*	Saos-2	^[152]^
Hedgehog pathway	Emodin	*Rheum palmatum L.*	MG-63	^[153]^
	Sauchinone	*Saururus chinensis (Lour.) Baill.*	U2OS, MG-63	^[154]^
	Galactoside	*Solanum nigrum L.*	U2OS, MG-63	^[155]^

JAK/STAT3 = tyrosine kinase/transcription factor 3, MAPK = mitogen-activated protein kinase, NF-κB = nuclear transcription factor-κB, Nrf2 = nuclear factor E2-related factor 2, OS = osteosarcoma, TCM = traditional Chinese medicine, TGF-β = transforming growth factor-β.

### 4.1. Treatment of OS by PI3K/Akt signaling pathway with TCM intervention

In terms of studies of monomers or active ingredients, Zhang et al^[84]^ found that alantolactone down-regulated the expression levels of MMP-2 and MMP-9 proteins by inhibiting the activity of PI3K/AKT signaling pathway, so as to inhibit the degradation and metabolism of extracellular matrix and reduce the invasion and migration of OS cells. Meanwhile, alantolactone inhibited this pathway, and it down-regulated the expression levels of CyclinD1 and Bcl-2 proteins, up-regulated the expression levels of p27, Bax, Cleaved-Caspase-3 and Cleaved-Caspase-8 proteins, so as to promote OS cell apoptosis as well as inhibit the growth of OS cells. Chelerythrine (CHE) is widely present in many herbs and is the main alkaloid component of Toddalia asiatica (L.) LAM with significant anti-tumor, anti-fungal, anti-inflammatory and anti-parasitic effects. Chen et al^[85]^ found that CHE significantly inhibited the stemness of OS cancer stem cell markers (including Nanog, Oct4 and nestin) by regulating the PI3K/AKT/mTOR signaling axis, and also decreased the expression of MMP-2/9 protein, so as to inhibit the malignant behavior of CSC. In addition, intervention with CHE also reversed the resistance of OS cells to chemotherapeutic agents (including doxorubicin, methotrexate and so on). Andrographolide, a diterpenoid lactone isolated from Andrographis paniculata, has been shown to have the activity of anti-tumor against several human cancer types. Huang et al^[86]^ found that andrographolide could also inhibit the activity of Wnt/β-catenin, PI3K/AKT and NF-κB signaling pathways in human-derived OS cells, and effectively inhibit the proliferation, migration and invasion of OS cells. Mickymaray et al^[87]^ experimentally found that emodin effectively down-regulated the PI3K/AKT/mTOR signaling cascade in MG-63 cells, and MG-63 cells treated with emodin showed accumulation of reactive oxygen species (ROS) in the cytoplasm, weakened MMP, increased the degree of nuclear damage and increased the number of apoptosis, suggesting that emodin is expected to be a chemotherapeutic agent for the treatment of human OS in the future.

In terms of the research of TCM compound, Bufei Decoction is composed of Radix Astragali, Radix Codonopsis, Schisandra chinensis, Rehmannia glutinosa, Aster and Morus alba, which has the effects of invigorating qi, activating blood circulation, strengthening qi and anti-cancer. Jiang et al^[156]^ experimentally found that Bufei Decoction significantly inhibited AKT, mTOR protein and mRNA expression, and inhibited the ability of OS cells to proliferate, invade and migrate by regulating PI3K/AKT/mTOR signaling pathway. Yiqi Sanyu Jiedu Decoction is composed of Radix Astragali, Heavy buildin and Coptis chinensis, which has the effects of clearing heat and detoxicating, removing blood stasis and dispersing spots, while Yin et al^[157]^ analyzed the potential mechanism of Yiqi Sanyu Jiedu Decoction in the treatment of OS with the help of network pharmacology and molecular docking technology, and the results showed that the active components in Yiqi Sanyu Jiedu Decoction might regulate multiple signaling pathways (including PI3K/Akt, MAPK, HIF-1 signaling pathways and so on) to play a therapeutic role in OS.

### 4.2. Treatment of OS by Wnt/β-catenin signaling pathway with TCM intervention

Qi et al^[88]^ experimentally found that piperine could inhibit the activity of Wnt/β-catenin signaling pathway and down-regulate the expression levels of MMP-2, VEGF protein, glycogen synthase kinase 3β (GSK-3β), β-catenin, target protein cyclooxygenase 2, cyclin D1 and C-myc protein, so as to effectively inhibit OS cell proliferation as well as promote OS cell apoptosis, and then inhibit the development of OS to some extent. Ren et al^[89]^ found that icariin could inhibit the activity of Wnt/β-catenin signaling pathway, down-regulate the expression levels of VEGF, MMP-9, p-GSK3β, p-β-catenin, C-myc and CyclinD1 protein, and promote the expression levels of Cleaved-caspase-3 protein, so as to inhibit the proliferation of OS cells and promote the apoptosis of OS cells. Wei et al^[90]^ experimentally found that isoquercitrin inhibited OS cell proliferation and metastasis, promoted OS cell apoptosis, and delayed the development of OS to some extent by inhibiting the Wnt/β-catenin signaling pathway and down-regulating the expression of β-catenin and its downstream targets C-myc, CyclinD1 and Survivin protein. Luce et al^[91]^ experimentally found that polydatin induced differentiation of human OS cells alone or in the presence of ionizing therapy by secreting sphingolipids and ceramides, suggesting that polydatin could serve as a potential candidate to assist in enhancing radiation-induced anticancer effects. Farnood et al^[92]^ pointed out that curcumin, as an effective anticancer compound, could improve the prognosis of OS patients by targeting and regulating the Wnt/β-catenin signaling pathway, but due to its poor hydrophobicity and bioavailability, it needs to be further explored in clinical practice.

Recent studies have found that lncRNAs and miRNAs play an important role in the regulation of various biological processes, such as inflammatory response, tumor cell proliferation, apoptosis and so on. As Zhang et al^[93]^ found that baicalein inhibited the activity of Wnt/β-catenin signaling pathway by promoting the expression of lncRNA-NEF, so as to induce apoptosis in OS cells. Interestingly, partial knockdown of lncRNA-NEF attenuated baicalein induced inhibitory effects on OS cell growth, metastasis and invasion, and successfully reversed inactivation of the Wnt/β-catenin signaling pathway, thus contributing to OS growth and metastasis. Xie et al^[94]^ found that allicin could inactivate the lncRNA MALAT1-miR-376a-Wnt/β-Catenin signaling pathway, thereby promoting oxidative stress and autophagy to inhibit the growth of OS. Hu et al^[95]^ experimentally found that cantharidin inhibited proliferation and metastasis of OS cells by down-regulating miR-214-3p, as well as reduced nuclear translocation of β-catenin by up-regulating DKK3, indicating that cantharidin may become a potential candidate for OS therapy by targeting miR-214-3p/DKK3/β-catenin signaling pathway.

Synergistic regulation with multiple mechanisms is also a unique advantage of active ingredients of TCM. Cassia seed, a Chinese herbal medicine, has been shown to have significant tumor killing effects, with cinnamaldehyde being the main active component. Huang et al^[96]^ found that cinnamaldehyde inhibited proliferation, migration, invasion and promoted apoptosis of OS cells by inhibiting Wnt/β-catenin and PI3K/Akt signaling pathways. Lycorine is an isoquinoline alkaloid, which found mainly in the corms of S. miltiorrhiza, and has been shown to inhibit the growth of many types of cancer. Yuan et al^[97]^ treated human OS cells with different doses of lycorine and found that the compound inhibited the growth of human OS cells by blocking Wnt/β-catenin, ERK1/2/MAPK and PI3K/Akt signaling pathways.

### 4.3. Treatment of OS by JAK/STAT3 signaling pathway with TCM intervention

Lu et al^[98]^ experimentally found that notoginsenoside R1 counteracted OS cell-mesenchymal stem cell-induced tumorigenesis in OS cells by blocking JAK2/STAT3 signaling pathway, decreasing OS cell viability and increasing Caspase-3/9 activity in cells, inhibiting inflammatory molecule IL-6 secretion, and regulating the function of OS and tumor microenvironment. Notably, administration with notoginsenoside R1 could also antagonize doxorubicin resistance in OS cells. Jiang et al^[99]^ experimentally found that polydatin induced autophagic death in human OS MG-63 cells by decreasing the expression and phosphorylation levels of STAT3 and increasing the expression of autophagy-related genes Atg12, Atg14, BECN1, and PIC3K3 through the STAT3 signaling pathway. Park et al^[100]^ found that hederacoside (HedC) increased the expression of p53, Bax and p21 proteins, but decreased the expression of Bcl-2 protein in OS cells, and HedC-mediated apoptosis was accompanied by a decrease in MAPK and STAT3 phosphorylation, indicating that HedC may play an anticancer role through intrinsic apoptosis and STAT3 signaling pathway in OS. Huang et al^[101]^ found that salidroside activated Caspase family-dependent intrinsic apoptosis pathway by inhibiting JAK2/STAT3 signaling pathway, so as to induce apoptosis of OS cells (MG63 and U2OS), while salidroside also inhibited cell growth and invasion, and then effectively inhibited the progression of OS.

In addition to glycosides can affect the growth process of OS, esters and alcohols rich in Chinese herbal medicines have also been gradually used in OS related studies. Agrimonia lactone is a lactone-containing steroid extracted from the traditional Chinese herbal medicine Agrimonia pilosa and has attracted much attention of researchers, because it has shown effective anti-proliferative activity in several cancer cell lines. Zhu et al^[102]^ found that agrimonia lactone activated the phosphatase activity of Src homology 2 phosphatase 2 (SHP-2), thereby inhibiting the JAK/STAT3 signaling pathway, helping to inhibit the proliferation of OS cells and promote the apoptosis of OS cells. Andrographolide has the effects of clearing heat and detoxicatin, reducing inflammation and relieving pain, while Cai et al^[103]^ found that this compound could reduce the phosphorylation level of STAT1/2 by inhibiting the activity of JAK2/STAT3 signaling pathway, reduce the release of pro-inflammatory cytokines or mediators (including TNF-α, IL-6, IL-1βand IL-10), so as to reduce the degree of paw swelling in mice, and block the progression of OS. Zhang et al^[104]^ found that paclitaxel could inhibit OS cell growth as well as induce OS cell apoptosis by mediating the SHP-1/JAK2/STAT3 signaling pathway, so as to delay the progression of OS to some extent. Resveratrol has shown effective antitumor therapeutic properties in various tumors. As Peng et al^[105]^ showed in vitro that resveratrol reduced cytokine synthesis through the STAT3 signaling, and inhibited JAK2/STAT3 signaling pathway, so as to inhibit OS cell proliferation and tumorigenesis ability.

With the continuous improvement of drug extraction technology, the complex components in Chinese herbal medicines have been gradually dissected out, and many unknown active components have also been used by researchers to treat OS. Annamalai et al^[106]^ treated OS MG-63 cells with a phytochemical called β-caryophyllene and found that β-caryophyllene regulated the JAK1/STAT3 signaling pathway in MG-63 cells through the mitochondrial apoptosis pathway induced by DNA fragmentation, and further promoted tumor cell apoptosis and inflammation. Ji et al^[107]^ found that sodium cantharidinate inhibited OS cell viability and migration by targeting STAT3 signaling pathway and induced OS cell apoptosis in vitro. Notably, combined treatment with sodium cantharidinate and EGFR inhibitor erlotinib enhanced growth inhibition in OS by preventing feedback activation of STAT3, suggesting that sodium cantharidinate is also able to target STAT3 signaling to eliminate EGFR inhibitor resistance in OS. Wang et al and Vundavilli et al^[108,109]^ all found that tanshinone I up-regulated the ratio of Bax to Bcl-2 protein by inhibiting the JAK/STAT3 signaling pathway, and inhibited the expression of matrix metalloenzymes (including MMP-2, MMP-9) protein associated with tumor invasion and migration, thereby delaying the growth and metastasis of OS.

### 4.4. Treatment of OS by Notch signaling pathway with TCM intervention

Cao et al^[110]^ experimentally found that cinobufotalin was able to inhibit Notch signaling pathway, decrease the expression of Notch-1, Hes-1, Hes-5 and Hey-1 genes in OS cell lines, inhibit OS cell survival and induce OS cell apoptosis, and then inhibit the growth of transplanted tumors in mice. Similarly, Farnood et al, Zahedipour et al^[92,111]^ applied curcumin to OS cells, and found that this compound could also downregulate Notch-1, Hes-1, Hey-1, and Hey-2 mRNA expression levels, inhibit cyclin D1, MMP-2, and MMP-9 protein expression levels, and regulate tumor cell growth, inflammatory expression, and apoptotic progression by inhibiting the Notch signaling pathway, so as to have the properties of anti-inflammatory, anti-oxidant, and anti-cancer. Niu et al^[112]^ experimentally found that cannabinoids down-regulated the expression of Notch-1, MMP-2 and VEGF through the Win/Notch signaling pathway, which could not only inhibit the generation of tumor collateral vessels, but also inhibit the proliferation, metastasis and invasion of OS cells. Qi et al^[113]^ experimentally found that dihydroartemisinin promoted OS cell apoptosis and inhibited the reproduction and growth of OS by inhibiting the signaling pathway activation of Notch1 and down-regulating the mRNA and protein expression levels of Notch-1, MMP-2, MMP-9 and Hes-1. Mei et al^[114]^ found that baicalein could inhibit the activity of Notch signaling pathway, up-regulate the expression level of pro-apoptotic protein Bax, and down-regulate the expression levels of anti-apoptotic proteins Bcl-2 and Hes1, thereby inhibiting the reproduction of OS cells and promoting the apoptosis of OS cells. Meanwhile, Mei et al^[115]^ also found that cordycepin inhibited the growth of OS cells by regulating the Notch signaling pathway and arresting the cell cycle in G0/G1 phase, while up-regulating the expression of Bax, Cleaved-Caspase-9 and Cleaved-Caspase-3 proteins, down-regulating the expression of Bcl-2, NICD1 and Hes1 proteins, thereby inducing the mitochondrial apoptosis pathway to induce apoptosis in OS cells.

Oleanolic acid is a naturally occurring triterpenoid that exhibits potential antitumor activity in several tumor cell lines. Xu et al^[116]^ found that oleanolic acid could interrupt the balance between pro-apoptotic and anti-apoptotic factors in OS cells by regulating Notch signaling pathway, thereby targeting mitochondrial pathway to induce apoptosis in OS cells. Pilose antler polypeptide is mainly composed of amino acids and is the main bioactive component of pilose antler, which has the effects of reliving swelling, reliving pain and trophic nerves. Lian et al^[117]^ found through relevant experiments that pilose antler polypeptide could inhibit the activity of Notch signaling pathway and down-regulate the expression levels of Notch1, Hes1, TNF-α, RIPK3 and MLKL proteins in OS cells, thereby inhibiting the reproduction of OS cells and promoting OS cell apoptosis.

### 4.5. Treatment of OS by TGF/Smad signaling pathway with TCM intervention

Sun et al^[118]^ experimentally found that oridonin could inhibit TGF-β-induced Smad2/3 phosphorylation, prevent Smad dimer translocation into the nucleus, and increase the expression of E-cadherin protein as well as decrease the expression of N-cadherin and Vimentin protein, thereby inhibiting the metastasis and invasion of OS cells by preventing EMT. Liu et al^[119]^ experimentally found that galangin enhanced the expression of osteoblast differentiation markers type I collagen, alkaline phosphatase, osteocalcin and osteopontin by inhibiting the TGF-β1/Smad2/3 signaling pathway, and increased alkaline phosphatase activity in human OS cells, thereby inhibiting the proliferation and growth of OS in vivo. Zhang et al^[120]^ experimentally found that hyperoside attenuated the phosphorylation level of downstream Smad2/3 by inhibiting the activity of TGF-β signaling pathway, and inhibited the proliferation of OS cells by arresting the G0/G1 phase of cell cycle, and promoted the differentiation of osteoblasts by up-regulating the expression levels of osteopontin, runt-related transcription factor 2 protein and osteocalcin. Kimura et al^[121]^ experimentally found that dihydroxycoumarin (aescinate or vuloxetine) inhibited M2 macrophage differentiation in tumor-associated macrophages and/or CyclinD1, CDK4, MMP-2, TGF-β1 and VEGF production in OS LM8 cells (in vitro) and tumor-bearing mouse models of high metastasis carrying LM8 cells (in vivo) through the TGF-β1 signaling pathway.

In addition to regulating TGF-β signaling pathway, the active ingredients in Chinese herbal medicines can synergize with other signaling pathways to affect the process of OS. Fox example, Motallebi et al^[122]^ found that naringenin synergistically inhibited the progression of OS (including apoptotic induction, cell cycle arrest and angiogenesis impairment in OS cells) by regulating various mechanisms, such as TGF-β, Wnt/β-catenin, PI3K/Akt and NF-κB signaling pathways. Dong et al^[123]^ experimentally found that the active components in safflower staghorn hoof grass up-regulated the expression level of E-cadherin protein and inhibited the production of Vimentin, Snail1 and Slug through various signaling pathways such as PI3K/Akt, ERK1/2 and Smad signaling pathways, so as to inhibit TGF-β2-induced EMT, which in turn inhibited the invasion and metastasis of human OS cells. Geranin, as an active compound isolated from geranium, is found to inhibit proliferation and induce apoptosis in tumor cells. As Wang et al^[124]^ found that geranin effectively inhibited the activity of PI3K/Akt and ERK1/2 signaling pathways, thereby inhibiting TGF-β1-mediated migration and invasion of human OS cancer cells due to the upregulation of MMP-9 protein.

### 4.6. Treatment of OS by NF-κB signaling pathway with TCM intervention

Hong et al^[125]^ found that diosgenin could inhibit OS growth in nude mice by inhibiting NF-κB signaling pathway, decreasing the expression of Bcl-2 protein, increasing the expression of Bax, Caspase-3, and Caspase-8 proteins. Wang et al^[126]^ experimentally found that pomegranate glycosides down-regulated the expression of p65, Survivin, XIAP, and CIAP2 proteins by inhibiting the activity of NF-κB signaling pathway, so as to inhibit angiogenesis as well as the growth and metastasis of OS, and promote OS cell apoptosis. Wang et al^[127]^ experimentally found that magnaine reduced the viability and invasion of OS cells by inhibiting the activation of NF-κB signaling pathway, while improved the resistance of OS cells to cisplatin. Jin et al^[128]^ experimentally found that teaflavin (TB) inhibited the growth cycle, migration, and invasion of human OS cells by inhibiting the NF-κB signaling pathway and down-regulating the expression of proteins, such as NF-κB, p-IKKα, p-IKKβ, E-cadherin, Vimentin, Snail-1, Slug and zinc finger E-box binding homeobox 1 (ZEB-1). Active ingredients in TCM can also affect the activity of NF-κB signaling pathway by regulating upstream targets. For example, Li et al^[129]^ found that asiaticoside reversed the malignant behavior of OS cells induced by polarization of M2 phenotype macrophages via decreasing the activity of TRAF6/NF-κB signaling pathway. In addition, Li et al^[130]^ found that magnolol decreased the expression of apoptosis-inhibiting and metastasis-related genes and up-regulated the expression of pro-apoptosis-related genes in human OS cells by inhibiting the MAPK/ERK/NF-κB signaling pathway, so as to inhibit the invasion and migration of OS cells and reduce the survival of OS cells. Similarly, Zhou et al^[131]^ found that paeonol could also reduce the activity of MAPK/NF-κB signaling pathway, inhibit solid tumor cell proliferation, metastasis and growth in OS mice, and promote apoptosis of OS cells. Radish thiophenol (SFE), a natural isothiocyanate found in cruciferous vegetables, has received increasing attention because of its anticancer effects in many cancers. Zhang et al^[132]^ used sulforaphane (SFE) to treat OS cells (U2OS and Saos-2) and showed that SFE reduced the activity of the FSTL1/NF-κB signaling pathway in a dose-dependent manner, thereby attenuating the proliferation, migration, and invasion of OS cells, and inducing apoptosis of OS cells.

The active ingredients in TCM can also cooperate with multiple mechanisms to regulate the occurrence and development of OS. For example, Guo et al^[133]^ demonstrated that curculigoside could hinder the growth, migration and invasion of OS cells in nude mice through JAK/STAT and NF-κB signaling pathways, and accelerate the apoptosis of OS cells. Chou et al^[134]^ found that dihydromyricetin (an aloe extract) inhibited migration of human OS cells by inhibiting SP-1 and NF-κB signaling pathways and regulating downstream urokinase plasminogen activator (uPA). Another report showed^[135]^ that fisetin down-regulated the expression levels of pEGFR, SOS-1, GRB2, Ras, PKC, p-ERK1/2, p-JNK, pp-38, VEGF, FAK, RhoA, PI3K, p-AKT, NF-κB, uPA, MMP-7, MMP-9 and MMP-13 proteins by regulating FAK, uPA and NF-κB signaling pathways, while promote the expression levels of GSK3β and E-cadherin proteins, which in turn inhibited the migration and invasion of OS cells. Moreover, Li et al^[136]^ found that ginsenoside Rh2 reducing the expression level of the anti-apoptotic factor (Bcl-2) and promoting the expression level of the pro-apoptotic factors (such as Bax, Caspase 3, and Caspase 9) by regulating NF-κB, MAPK and PI3K/Akt/mTOR signaling pathways in OS cells, so as to hinder the proliferation and migration of OS cells and induce their apoptosis.

### 4.7. Treatment of OS by MAPK signaling pathway with TCM intervention

Different extracellular stimuli (such as the intervention of TCM components) can influence different MAPKs signaling pathways and mediate different biological responses through their mutual regulation. The MAP kinases of mammalian can be divided into 3 classes based on their structure and function: stress-activated protein kinases or JNKs. ERKs. p38 MAPK. In recent years, more and more studies have found that MAPKs signaling family mediated by active ingredients of TCM plays an important role in the treatment of OS.

Zheng et al^[137]^ found that diosgenin inhibited the proliferation, migration and invasion of OS cell, and induced OS cell apoptosis by up-regulating ROS-mediated P38/MAPK signaling pathway. Similarly, studies by Ning L, Yuan XH et al^[97,138]^ found that lycorine was also able to increase the intracellular ROS content, thereby activating the p38-MAPK signaling pathway and inhibiting the expression level of the downstream member p53 protein, which in turn arrested the growth of mouse OS cells in G1 phase, and promoted apoptosis of mouse OS cells. Delphinidin is a natural phytoestrogen with structures similar to estrogen that can inhibit cell proliferation and induce apoptosis in a variety of tumors. As Tian et al^[139]^ found delphinidin up-regulated the expression of pro-apoptotic proteins Caspase-3, Caspase-9 and Bax by inhibiting the phosphorylation of the p38/MAPK signaling pathway, and decreased the expression of the anti-apoptotic protein Bcl-2, thereby reducing the viability of human OS cells, increasing the degree of MMP loss, and inducing apoptosis in OS cells. When OS cells were treated with apocynin, Yong et al^[140]^ found that the p38-MAPK signaling pathway was also activated in OS cells, and down-regulated the expression of Bcl-2 protein and up-regulated the expression of Bax, Cleaved-Caspase-3, Cleaved-Caspase-8 and Cleaved-Caspase-9 proteins, so as to promote the apoptosis of OS cells. Vundavilli, Hu et al’s experiments^[109,141]^ all found that tanshinone could mediate the expression of Src protein, a downstream member, through MAPK/ERK signaling pathway, thereby inducing OS cell death. Fangji, as a TCM, is commonly used to treat silicovolcanoconiosis, hypertension and arthritis, and has now been confirmed by many studies to have effective anti-tumor growth properties, while tetrandrine is extracted from the rhizomes of Fangji. Xu et al^[142]^ experimentally found that lentinan could target and reduce the phosphorylation levels of MAPK and ERK proteins on the MAPK/ERK signaling pathway by up-regulating the expression of microRNA-340 in human OS MG63 cells, thereby reducing OS cell proliferation and inducing their apoptosis and autophagy.

Xu, Gao et al^[143,144]^experimentally all found that berberine down-regulated the expression of MMP-2, MMP-9 and Bcl-2 proteins by regulating the MAPK/JNK signaling pathway, while up-regulating the expression of Bax protein, thereby significantly inhibiting the growth of OS in nude mice as well as attenuating the migration and invasion of OS cells. Myricetin is an antioxidant, which found in berries, nuts, tea, wine and vegetables. Park et al^[145]^ used myricetin to treat canine OS D-17 and DSN cells, and they found that the drug could regulate the MAPK signaling pathway and keep the downstream member JNK in a phosphorylated state, thereby inducing DNA fragmentation, disrupting redox homeostasis and mediating mitochondrial membrane potential loss, and ultimately promoting OS cell apoptosis, suggesting that myricetin may be a potentially effective and less toxic therapeutic agent to prevent and control the progression of canine OS.

Studies have shown that the active ingredients rich in TCM can also synergistically regulate the progression of OS with the characteristics of “multiple targets and multiple mechanisms.” Fox example, Kang et al^[158]^ experimentally found that delphinidin inhibited epithelial-mesenchymal transition in OS cells by regulating the phosphorylation levels of ERK and p38, downstream members of the MAPK signaling pathway, thereby reducing the migration and invasion of OS cells, and promoting OS cell apoptosis. Ryu et al^[146]^ found that quercetin inhibited the expression levels of AKT, P70S6K, and S6 proteins by regulating PKB and MAPK signaling pathways, up-regulating the phosphorylation levels of ERK1 or p38, c-Jun N-terminal kinase, and P90RSK proteins, thereby changing the growth cycle, mitochondrial depolarization, reactive oxygen species levels, and cytosolic calcium concentrations of OS cells, which in turn decreased the proliferative characteristics of OS cells as well as increased programmed cell death in OS cells. Cardamon, as a Chinese herbal medicine, which is rich in cardamonin, has some inhibitory effects on various tumors. In the Zhang et al’s study,^[147]^ cardamonin was found to initiate MAPK signaling pathways and further increase phosphorylation levels of p38 and JNK, thereby inducing OS cells arrest in G2/M phase and inhibiting OS cells growth in xenograft mouse models, while reducing the migration and invasion of OS cells.

### 4.8. Treatment of OS by other signaling pathways with TCM intervention

Active components extracted from Chinese herbal medicines can also intervene other signaling pathways (such as Nrf2, Hippo/YAP, RANK/RANKL/OPG, Hedgehog and so on) to affect the development of OS. As Xie et al’s study^[148]^ found tanshinone IIA inhibited proliferation and metastasis of OS MG63 cells by decreasing the transcription and activity of AMPK and Nrf2 through regulating AMPK/Nrf2 signaling pathway. Lu et al^[149]^ experimentally found that oridonin induced mitochondria-mediated apoptosis by activating PPAR-γ and inhibiting the Nrf2 signaling pathway, increasing the ratio of Bax to Bcl-2, damaging the MMP protein, triggering the ROS generation and promoting the cleavage of Caspase-3/9, so as to inhibit the development of OS. Yan et al^[150]^ experimentally found that emodin could cause a decrease in the content of ROS, the translocation of Nrf2 and the driven luciferase activity of ARE in OS cells by inhibiting the activity of Nrf2 signaling pathway, thus OS MG63 cells were protected from cisplatin-induced oxidative stress injury. Qin et al^[151]^ experimentally found that norcantharidin not only inhibited the proliferation and metastasis of OS cells, but also promoted OS cells apoptosis by regulating the Hippo/YAP signaling pathway. Zaklazos-Szyda et al^[152]^ experimentally found that Jialien up-regulated the mRNA expression levels in Runt-related transcription factor 2, ALP, collagen 1 and osteonectin by regulating RANK/RANKL/OPG and NF-κB signaling pathways, while the ratio of NF-κB ligand to osteoprotegerin (RANKL/OPG) decreased, and the secretion of inflammatory factors (IL6 and TNFα) down-regulated, thereby inhibiting the growth of OS cells. Qu et al^[153]^ experimentally found that emodin inhibited the viability and radioresistance of OS cells as well as promoted OS cell apoptosis by regulating the Sonic Hedgehog signaling pathway. Zhou et al^[154]^ experimentally found that tribaicalein inhibited hypoxia-induced OS cell invasion and EMT by inactivating the Sonic Hedgehog signaling pathway, while promoting OS cells apoptosis. Zhao et al^[155]^ experimentally found that galactoside inhibited the proliferation, growth, migration and invasion of OS cells, promoted OS cells apoptosis through the GSK3βinactivation mediated Hedgehog/Gli1 signaling pathway.

## 5. Ferroptosis-related signaling pathway and OS

**Figure 2.**
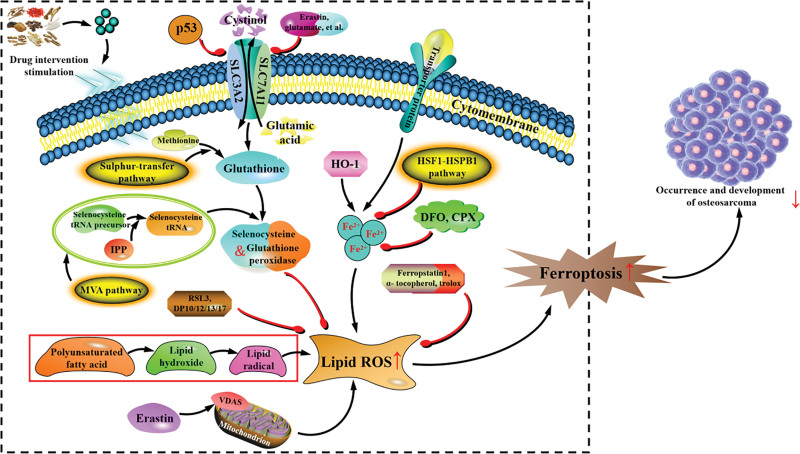
The mechanism of ferroptosis on the occurrence and development of OS. OS = osteosarcoma.

Ferroptosis is different from apoptosis, pyroptosis, autophagy and other programmed cell death, which belongs a programmed cell death caused by iron ion-dependent intracellular lipid peroxide accumulation, and iron ions of peroxides can promote ferroptosis through Fenton reaction peroxides. Increasing evidence suggests that ferroptosis is involved in various physiological and pathological processes, particularly the development of tumors.^[159]^ Modern medical research has found that tumor cells are very sensitive to ferroptosis, and the regulation of ferroptosis inducer or ferroptosis-related genes can not only inhibit tumor growth, but also improve the drug resistance of tumor cells.^[160]^ Moreover, the ferroptosis inhibitory protein can participate in the reduction of CoQ10, and the resulting CoQ10H2 can capture lipid peroxidation free radicals and inhibit ferroptosis by preventing the accumulation of intracellular lipid peroxides. At present, a large number of studies suggest that there is a close relationship between ferroptosis and tumor immune response, and some small molecule drugs based on ferroptosis pathway have shown good efficacy in the field of cancer immunotherapy, so as to provide a new direction for the research and development of cancer drugs.^[161–164]^ A series of studies have also shown that ferroptosis-related inducers can synergize radiation and chemotherapeutic drugs (such as cisplatin) to enhance the sensitivity of tumors for chemoradiotherapy,^[165,166]^ which in turn provides new ideas for clinical treatment strategies (Fig. 2).

### 5.1. Role and potential mechanism of ferroptosis in OS

Ferroptosis is a programmed cell death form dependent on the concentration of iron ion and closely related to excessive lipid peroxides, which can not only target and regulate key molecules (such as GPX4 or System Xc-) to induce iron death in tumor cells, but also target and regulate the oxidative level of iron ion (FINO2) or the overload od intracellular iron ion (FeCI2) to induce ferroptosis in tumor cells. With the deepening of research, it has been found that the molecular mechanism of ferroptosis is mainly initiated by 2 pathways, of which the intrinsic pathway triggers ferroptosis by blocking the activation of intracellular anti-oxidant enzymes, while the extrinsic pathway triggers ferroptosis by inhibiting cell membrane transporters or activating multiple iron transporters.^[167]^

Studies have demonstrated that ferroptosis are different from various other forms of death in that it is quite different in morphology, biological characteristics, immunological characteristics, and genetic characteristics. In terms of biochemical characteristics, it is closely related to the accumulation of lipid oxides and ROS, metabolism of cystine/glutamate reverse transporter (a cystine/glutamate reverse transporter, System Xc-), release of key factors (such as STAT3, Nrf2, GPX4, MEK, p53 and so on), and transduction of Nrf2/GPX4 signaling.^[168]^ In addition, it has been experimentally demonstrated that nano-formulations can increase the concentration of iron ion and the levels of lipid reactive oxygen species (ROS) in tumor cells, which triggers the Finton reaction, so as to react with intracellular unsaturated fatty acids to produce lipid peroxides and ultimately induces tumor cells to move towards death.^[169]^ Recent studies have also identified that MicroRNAs and long noncoding ribonucleic acids (lncRNAs) are critical mediators regulating ferroptosis.^[170]^

### 5.2. Morphological characteristics of ferroptosis

Ferroptosis, as a novel mode of cell death, is identified in recent years that distinguishes apoptosis, necroptosis, and autophagy. Meanwhile, ferroptosis is an oxidative cell death that occurs under the induction of small molecules and is dependent on the iron ion. To distinguish ferroptosis from other modes of programmed death, we can identify it based on its ultrastructural features. Under the field of transmission electron microscope, the cell membrane will appear fragmentation and blebbing phenomenon, the volume of mitochondria gradually shrinks and becomes smaller, the mitochondrial ridge decreases or even disappears, the density of mitochondrial membrane increases, but the morphology of the nucleus is normal.^[171]^

### 5.3. Accumulation of lipid oxides and ROS is necessary for ferroptosis in tumor cells

Lipid peroxidation is necessary for the development of ferroptosis. Lipid peroxides are important regulators in the development of ferroptosis, and can cause damage to phospholipid bilayers on cell membranes, ultimately leading to ferroptosis in cells. Its specific development process refers to the lipid peroxidation of intracellular ROX in combination with the propylene group on polyunsaturated fatty acids (polycloud-saturated fatty acids and PUFAs), which converts excessive intracellular PUFAs into lipid oxides and peroxides, ultimately leading to the destruction of cell membrane structure.^[172]^ It has been demonstrated^[173]^ that phosphatidylethanolamines (PEs) of arachidonic acid (AA) or its derivative epinephrine are key phospholipids that induce ferroptosis in cells, and the contents of AA and PEs in cells will be significantly reduced when cells are treated with the ferroptosis inducer Erastin. Toxic lipid peroxidation products (such as 4-hydroxy-2-acrylic acid, malondialdehyde and so on) will be formed during lipid peroxidation, which can irreversibly destroy lipids on the cell membrane, so as to change in the structure and function of cell membrane, cytoskeletal rupture, and ultimately cause the occurrence of ferroptosis.^[174]^

### 5.4. Induction of ferroptosis in tumor cells by inhibition of cystine/glutamate reverse transporter (a cystine/glutamate reverse transporter, System Xc-)

System Xc- is a receptor with cystine/glutamate as a transporter widely distributed on human cell membranes, which is a component of an important antioxidant system in cells and is a heterodimer composed of 2 subunits SLC7A11 and SLC3A2. When System Xc is inhibited, the exchange of cystine is also inhibited, thus affecting the synthesis of glutathione, resulting in reduced activity of GPX, decreased cellular antioxidant capacity, excessive accumulation of lipids and ROS in the cytoplasm, and ultimately oxidative damage and ferroptosis in cells. It has been found^[170,175]^ that ferroptosis-related activators (such as Erastin, sulfasalazine) can reduce GSH synthesis by inhibiting the activity of System Xc − and reducing cystine uptake by cells, so as to induce ferroptosis in cells. Moreover, p53 can also directly or indirectly down-regulate the expression of System Xc-subunit SLC7A11 to inhibit the uptake of cystine by System Xc-, so as to seriously affect the activity of GPX4, resulting in significantly reduced antioxidant capacity of cells, excessive accumulation of lipids and ROS in the cytoplasm, and ultimately causing the occurrence of ferroptosis.^[176–178]^

### 5.5. Induction of ferroptosis in tumor cells by reducing GPX4 activity

Critical proteins on the ferroptosis signaling pathway can be targeted by drugs, such as glutathione peroxidase 4 (GPX4), which is one of the key enzymes in the regulation of ferroptosis in cells and plays an important role in the development of ferroptosis. GPX4, as the core of iron death, is able to convert lipid peroxides into lipid alcohols, which in turn attenuates lipid peroxide-induced cytotoxicity, while inhibition of GPX4 activity promotes lipid peroxide accumulation which in turn induces iron death.^[179,180]^ Under normal physiological conditions, GPX4 is able to directly convert reduced glutathione (GSH) into oxidized glutathione (GSSG), while it is able to reduce cytotoxic lipid peroxides (R-OOH) to non-cytotoxic alcohols (R-OH). When GPX4 activity is inhibited in cells, they are more sensitive to ferroptosis. Conversely, when the expression of GPX4 activity is promoted, cells can become tolerant to the occurrence of ferroptosis. It has been found^[181,182]^ that RSL3, as an iron death inducer, can effectively act on GPX4 in cells and directly inhibit GPX4 activity, so as to reduce the antioxidant capacity of cells, allow a large amount of ROS to accumulate in cells, and ultimately lead to the occurrence of ferroptosis. In addition, certain compounds (such as ML162, withaferinA, DPI7, DPI10) and the chemotherapeutic agent (such as altretamine) can also directly or indirectly inhibit the expression of GPX4 activity in cells and ultimately induce ferroptosis in cells.

### 5.6. Treatment of OS with TCM intervening ferroptosis signaling pathway

Although there is no authoritative evidence-based medical evidence to support TCM in clinical practice, according to TCM syndrome differentiation and decoction, it can quickly relieve symptoms, improve the condition, and has less adverse reactions, thus showing unique advantages. Increasing studies have shown that TCM can hinder the development of OS by targeting the signaling cascade of ferroptosis. Luo et al^[183]^ showed that flavonoids (Psoralea corylifolia) regulated ferroptosis signaling pathway through STAT3/P53/SLC7A11 axis, up-regulated the expression levels of transferrin receptor, divalent metal transporter-1 and p53 protein, down-regulated the expression levels of ferritin light chain, ferritin heavy chain, p-STAT3 (705), SLC7A11 and glutathione peroxidase-4 protein, so as to promote ferroptosis in OS cells. Similarly, experiments by Tang et al^[167]^ showed that ursolic acid degrades ferritin by activating autophagy and inducing intracellular ferrous ion overload, which leads to ferroptosis in tumor cells, and synergistically enhances the DNA damage effect of cisplatin on OS cells. Salaroli, Isani et al’s experiments^[184,185]^ all found that hydroalcoholic extract of Artemisia annua has anticancer activity against canine OS cell lines via the ferroptosis signaling pathway. Lin Hai et al^[186]^ pointed out that the curcumin analogue EF24 is an inducer of ferroptosis and is able to mediate ferroptosis in OS cells, and dose-dependently up-regulate the expression level of HMOX-1 protein in OS cells, which in turn promotes apoptosis in OS cells.

## 6. Summary and future perspectives

In summary, Chinese herbal medicines downregulate the release and activity of inflammatory factors, reduce oxidative stress, regulate abnormal energy metabolism, improve tumor microenvironment, induce autophagic death, inhibit cell proliferation, differentiation and metastasis, promote apoptosis, and ultimately achieve the purpose of inhibiting the development of OS by regulating the transduction of signaling pathways, such as PI3K/Akt, Wnt/β-catenin, JAK/STAT3, Notch, TGF-β/Smad, NF-κB, MAPK, Nrf2, GPX4, Hippo/YAP, OPG/RANK/RANKL, Hedgehog, ferroptosis and so on. As a gift from nature, Chinese herbal medicine plays an indispensable role in the history of human health development, and it has the characteristics of multiple activities, multiple components, multiple targets and multiple pathways, while it has therapeutic advantages, such as significant efficacy and less side effects. A large number of studies have shown that the active ingredients rich in Chinese herbal medicines can effectively inhibit the reproduction and differentiation of OS cells and promote OS cell apoptosis, and even some compounds can reverse the acquired resistance of OS cells to chemotherapeutic drugs. In view of these researched backgrounds, this review mainly elaborates on the process of inhibiting OS from the intervention of related signaling pathways by TCM, which not only aims to provide reference and ideas for clinicians in the treatment of OS, but also provide a theoretical basis for scientific researchers in the development of OS drugs.

However, there are still many problems and difficulties to be conquered urgently in the current study. At present, the monomers, active ingredients and acted mechanism of TCM have not been thoroughly studied and clarified, and TCM plays an auxiliary role in the clinical treatment of OS and has not become the main treatment. More importantly, there is a lack of effective connection between clinical practice and basic research, and the main research on OS focuses on single herbs and active ingredients, rarely involving TCM compounds. Of course, because multiple targets and multiple ways will inevitably lead to its complex mechanism of action, which also greatly increases the difficulty of scientific research work, and the characteristics of multiple targets and multiple ways possessed by TCM are similar to the overall concept of TCM. Therefore, it is still very necessary to involve the study of TCM compounds. In addition, although most current studies have revealed that TCM has a significant effect on the treatment of OS animal experiments and related cell experiments, there is a lack of systematic, large-sample clinical trials under the guidance of evidence-based medicine. Meanwhile, it is still necessary to use a combination of multi-omics (including genomics, epigenomics, proteomics, transcriptomics, microbiomics and metabolomics and so on) and multi-science to deeply encode RNA and non-coding RNA in the future, so as to directly or indirectly mediate the treatment of OS by related signaling pathways. In addition, it is necessary to give full play to the characteristics and advantages of network pharmacology of TCM, so as to excavate more therapeutic drug targets with exact efficacy and further promote the application of TCM in the treatment of OS.

Notably, ferroptosis plays a very important role in the process of OS. Specifically, ferroptosis can not only make cysteine synthesis GSH inactive, iron-dependent lipid oxides and abnormal accumulation of ROS accumulated, but also can inhibit STAT3/Nrf2/GPx4 signaling pathway, so as to induce ferroptosis in OS cells, which is of great significance in the treatment of malignant tumors in clinical practice.

## Author contributions

**Conceptualization:** Hongzhuan Li.

**Formal analysis:** Yanqiang Li, Xiaoshou Zhang, Lijun Wang, Yasai Yao, Baohong Zhu.

**Funding acquisition:** Xiping Chai, Xingrong Hu, Bangneng Zhang.

**Writing – original draft:** Yuezhen Liu, Bing Jiang.

**Writing – review & editing:** Hengwei Shi, Xiping Chai, Xingrong Hu, Bangneng Zhang.
